# Effects of cognitive emotion regulation strategies on mood and cortisol in daily life in women with premenstrual dysphoric disorder

**DOI:** 10.1017/S0033291722002495

**Published:** 2023-08

**Authors:** Sibel Nayman, Theresa Beddig, Iris Reinhard, Christine Kuehner

**Affiliations:** 1 Research Group Longitudinal and Intervention Research, Department of Psychiatry and Psychotherapy, Central Institute of Mental Health, University of Heidelberg, Medical Faculty Mannheim, Mannheim, Germany; 2 Department of Biostatistics, Central Institute of Mental Health, University of Heidelberg, Medical Faculty Mannheim, Mannheim, Germany

**Keywords:** ambulatory assessment, cortisol, emotion regulation, menstrual cycle, premenstrual dysphoric disorder

## Abstract

**Background:**

The psychological risk factors of premenstrual dysphoric disorder (PMDD) are not fully understood, but initial evidence points to a potential role of unfavorable cognitive emotion regulation (ER-) strategies. Given the symptom cyclicity of PMDD, ambulatory assessment is ideally suited to capture psychological and physiological processes across the menstrual cycle. Our study examines habitual ER-strategies in women with PMDD and their predictive value for the course of mood and basal cortisol across the cycle in affected women.

**Methods:**

Women with and without PMDD (*n* = 61 each) were compared regarding habitual mindfulness, reappraisal, and repetitive negative thinking (RNT). Momentary affect and cortisol output were assessed over two consecutive days per cycle phase (menstrual, follicular, ovulatory, late luteal).

**Results:**

Women with PMDD reported lower mindfulness, less use of reappraisal and stronger RNT than controls (*p*s < 0.035). In women with PMDD, higher mindfulness and reappraisal and lower RNT predicted decreased negative and increased positive affect across the menstrual cycle (*p*s < 0.027). However, women using more favorable ER-strategies displayed stronger mood cyclicity, resulting in stronger mood deterioration in the late luteal phase, thereby resembling women with more unfavorable ER-strategies toward the end of the cycle. Lower mindfulness predicted lower cortisol in the menstrual phase.

**Conclusions:**

Protective ER-strategies seem to be generally linked to better momentary mood in women with PMDD, but do not appear to protect affected women from premenstrual mood deterioration. Habitual mindfulness, in turn, seems to buffer blunted cortisol activity in women with PMDD, especially in the menstrual phase.

Premenstrual dysphoric disorder (PMDD) is characterized by key affective and further psychological, behavioral, and physiological symptoms, which occur during the late luteal phase of the menstrual cycle and remit within the week following menses [American Psychiatric Association (APA), 2013]. PMDD affects around 5% of women of fertile age (Beddig & Kuehner, 2017; Eisenlohr-Moul, 2019) and causes clinically significant distress or functional impairment in daily life (APA, 2013) with increased risk of a chronic symptom course (Wittchen, Becker, Lieb, & Krause, 2002) and suicidality (Osborn, Brooks, O'Brien, & Wittkowski, 2020). PMDD must be differentiated from the more frequent and less severe premenstrual syndrome (PMS), which is not uniformly defined and does not necessarily require affective symptoms (Hantsoo & Riddle, 2021).

Research on PMDD risk factors has mainly focused on its pathophysiology, suggesting a hypersensitivity to normal fluctuations of reproductive steroid hormones, which interacts with the GABAergic, serotonergic, and HPA-axis systems (Beddig & Kuehner, 2017; Eisenlohr-Moul, 2019; Hantsoo & Epperson, 2015). Regarding the latter, research on PMDD suggests that higher levels of perceived stress are linked to exacerbations of premenstrual symptoms (e.g. Gollenberg et al., 2010; Namavar Jahromi, Pakmehr, & Hagh-Shenas, 2011; Owens & Eisenlohr-Moul, 2018), pointing to a possible role of HPA-axis dysfunction in PMDD (Kiesner & Granger, 2016; Owens & Eisenlohr-Moul, 2018). However, respective evidence is mixed, ranging from blunted to increased cortisol activity in women with premenstrual disorders (e.g. Huang, Zhou, Wu, Wang, & Zhao, 2015; Kiesner & Granger, 2016; Owens & Eisenlohr-Moul, 2018). The missing differentiation between PMDD and PMS in previous research may be one reason for these inconsistencies. For example, Odber, Cawood, and Bancroft (1998) identified higher premenstrual basal cortisol levels compared to postmenstrual levels in women with mild PMS symptoms, but demonstrated a reverse cortisol pattern with significantly decreased basal cortisol levels in the premenstrual phase for women with severe premenstrual mood changes. Similarly, experimental studies point to hypoactivation of the HPA-axis in response to stress in women with PMDD (Huang et al., 2015; Klatzkin, Lindgren, Forneris, & Girdler, 2010). Reduced HPA-axis activation, in turn, has been shown to be associated with poorer mental and physical health, reflecting a biological mechanism of a spectrum of stress-related disorders (e.g. Adam et al., 2017).

Multifactorial approaches emphasize the additional role of intrapersonal psychological factors, which interact with possible biological characteristics in PMDD (Blake, 1995; Kleinstäuber et al., 2016). In this regard, the high lifetime comorbidity of PMDD with depressive and anxiety disorders (Cohen et al., 2002; Yen et al., 2020) point to the potential role of transdiagnostic psychological risk factors in the development and maintenance of PMDD. In particular, the role of possible cognitive emotion regulation (ER) dysfunction has been discussed (Owens & Eisenlohr-Moul, 2018). According to strategy-based models, ER-strategies can be classified in terms of their formal characteristics, such as being adaptive *v.* maladaptive (Naragon-Gainey, McMahon, & Chacko, 2017). When used habitually, adaptive and maladaptive ER-strategies have been shown to be linked to favorable *v.* unfavorable long-term psychological and physiological health outcomes (McRae & Gross, 2020).

Initial evidence indicates that women with PMDD tend to use more maladaptive strategies, such as avoidance coping, catastrophizing, and ruminative strategies (Craner, Sigmon, Martinson, & McGillicuddy, 2014; Craner, Sigmon, & Young, 2016; Eggert, Kleinstäuber, Hiller, & Witthöft, 2017). In particular, habitual rumination has been shown to contribute to larger increases in premenstrual depressive symptoms in women with premenstrual mood disorders (Dawson et al., 2018). In turn, habitual reappraisal (Wu, Liang, Wang, Zhao, & Zhou, 2016) and mindfulness (Lustyk, Gerrish, Douglas, Bowen, & Marlatt, 2011) were linked to less premenstrual symptom severity in nonclinical samples.

Since neuroticism was found to be positively associated with PMDD (Aperribai, Alonso-Arbiol, Balluerka, & Claes, 2016; Miller et al., 2010) and higher neuroticism may foster the use of more maladaptive ER-strategies (Yang et al., 2020), it is essential to take neuroticism scores into account when studying the use of habitual ER-strategies in PMDD. The same applies to clinical symptom levels, such as concurrent depression scores, which may affect the assessment of the habitual use of certain ER-strategies.

Previous PMDD research has mainly studied the role of habitual ER-strategies in cross-sectional study designs (e.g. Craner et al., 2014; Lustyk et al., 2011). However, only few studies have investigated their effects on cycle-related symptom change (e.g. Craner et al., 2016; Dawson et al., 2018). Furthermore, to our knowledge, studies investigating associations of ER-strategies with cortisol activity across the menstrual cycle in women with PMDD are totally lacking.

Given the cyclicity of PMDD symptomatology, ambulatory assessment (AA) designs with multiple real-time assessments during daily life are well-suited (Bosman, Jung, Miloserdov, Schoevers, & Aan Het Rot, 2016; Owens & Eisenlohr-Moul, 2018). AA allows to capture within-person psychological and physiological processes across the menstrual cycle, reduces recall biases and increases ecological validity (Trull & Ebner-Priemer, 2013). In this context, our previous research provided clear evidence of menstrual cycle-related variations of daily life experiences in women with PMDD compared to healthy controls (Beddig, Reinhard, Ebner-Priemer, & Kuehner, 2020; Beddig, Reinhard, & Kuehner, 2019). In particular, women with PMDD showed increased negative and decreased positive affect (PA) (Beddig et al., 2020) and increased psychological stress-reactivity during the late luteal phase as well as a delayed cortisol awakening response and flattened diurnal cortisol slope across the menstrual cycle compared to healthy controls (Beddig et al., 2019). Moreover, high negative affect (NA) and low cortisol output independently predicted a worse clinical course of PMDD symptomatology over a 4-month interval (Beddig & Kuehner, 2020).

## The current study

In the present AA study, we aimed to examine the role of habitual ER-strategies for the cyclical course of momentary mood and cortisol activity in women with PMDD. Consistent with previous research, we expected that women with PMDD would report lower levels of mindfulness and reappraisal and higher levels of repetitive negative thinking (RNT) compared to controls. We further expected that in women with PMDD, lower levels of mindfulness and reappraisal and higher levels of RNT would predict (a) increased NA, (b) decreased PA, and (c) decreased cortisol activity, especially in the late luteal phase compared to women with more favorable ER-strategies. Finally, we examined whether observed associations would hold when controlling for levels of neuroticism and depressive symptoms.

## Methods

### Participants

The present sample and study design have been previously described in detail in Beddig et al. (2019). Women with and without PMDD (*n* = 61 each) were recruited at the Central Institute of Mental Health (CIMH), Mannheim, Germany between March 2016 and October 2018. Inclusion criteria included (a) age between 20 and 42, (b) consistent length of menstrual cycle between 22 and 34 days, and (c) fulfillment of diagnostic criteria of a PMDD diagnosis based on DSM-5 criteria (PMDD group) or exemption from any PMDD affective core symptoms (control group). Women were ineligible if they were pregnant or lactating during the last 6 months, taking hormonal contraceptives and pharmaceutical medication during the last 3 months, or if they reported a body mass index <18 or >35, late evening or night shifts, a history of gynecological diseases (e.g. hysterectomy or ovariectomy), psychotic or bipolar disorder, and current substance abuse or dependence. The study protocol was approved by the ethics committee of the Medical Faculty Mannheim, Heidelberg University. All participants gave written informed consent and were paid 100€ for their participation.

### Procedure

The procedure consisted of a preliminary telephone screening, a baseline session, and 8 days of subsequent AA. Demographic and clinical characteristics, inclusion and exclusion criteria, as well as psychological traits (i.e., habitual ER-strategies; see below) were assessed during the baseline session at the CIMH in Mannheim (Germany). Eligible participants then received a study smartphone and detailed instructions regarding the AA procedure.

To verify the diagnosis of PMDD, the SCID-PMDD, a reliable Structured Interview for DSM-IV-TR PMDD (*κ* = 0.96; Accortt, Bismark, Schneider,& Allen, 2011) was administered to both samples during the diagnostic baseline session. The SCID-PMDD covers all symptom criteria of PMDD together with the criterion of relational, occupational, and recreational impairment or distress and the exclusion criterion of a mere exacerbation of symptoms of another disorder (cf. Kuehner & Nayman, 2021). The interview format for 11 symptoms of PMDD is modeled after the Structured Clinical Interview for DSM-IV for Axis I (SCID-I; Wittchen, Zaudig, & Fydrich, 1997), with additional questions on the timing of symptom-on- and offset across the menstrual cycle and the number of symptomatic cycles experienced for each of the 11 symptoms. A diagnosis of PMDD required fulfilling respective criteria within the diagnostic algorithm adapted for DSM-5, according to which functional impairment as a criterion is not mandatory if the premenstrual symptoms are associated with clinically significant distress (APA, 2013). Controls had to be free of any PMDD affective core symptom. In order to keep the compliance rate high and to avoid further participant burden within the AA-design, additional prospective daily symptom ratings during at least two symptomatic cycles before study inclusion were not required. Current and lifetime DSM-IV-TR Axis I psychiatric comorbidities and exclusion criteria were assessed with the SCID-I (Wittchen et al., 1997). All interviews were performed by a trained research psychologist (T.B.).

### Ambulatory assessment (AA)

AA was carried out using *Motorola Moto G 2nd Generation* smartphones with the software *movisensXS, version 0.6.3658* (movisens GmbH, Karlsruhe, Germany). AA took place over two consecutive days per menstrual cycle phase (menstrual, follicular, ovulatory, and late luteal phase).

The typical menstrual cycle lasts about 28 (21–35) days and can be divided into four cycle phases with predictable fluctuations of the ovarian hormones *progesterone* (P4) and *estradiol* (E2). It starts with the onset of menstruation, which represents the cycle day 1, endures about 5 days and is characterized by low P4 and E2 levels. The follicular phase is marked by consistently low P4 and rising E2 levels with a peak prior to ovulation, which is followed by a rapid E2 decrease after ovulation. The luteal phase covers the days from ovulation until menses during which E2 and P4 gradually rise, reaching their highest levels during the mid-luteal phase, and then show a rapid withdrawal during the late luteal (premenstrual) phase, i.e., the week prior to the next menses (Schmalenberger et al., 2021).

Individual cycle calendars were prepared based on the date of the last menstruation onset and the average cycle length in order to specify the start date of ovulation testing and exact days of the AA. The ovulation phase was identified by a chromatographic ovulation test (gabControl hlH Ovulationsteststreifen, gabmed, Cologne). The testing started a few days before the predicted ovulation and had to be continued until a positive result occurred. If ovulation did not occur, women were asked to repeat the testing in the next menstrual cycle. In order to avoid sequence effects, they started AA in different cycle phases.

The *menstrual phase* was assessed on the second and third days of menstruation (*M* = 2.95 days, s.d. = 2.21). The assessments during the *follicular phase* were examined on the second and third days after the end of menstruation (*M* = 8.61 days, s.d. = 1.94). The *ovulatory phase* (*M* = 17.15 days, s.d. = 2.0) was assessed on the 2 days following a positive ovulation test result. In case of a negative test, participants were asked to repeat the testing during the following menstrual cycle. Assessments of the *late luteal phase* took place on the fourth and third day before the next expected menstruation (*M* = 26.38 days, s.d. = 3.02). If the menstruation occurred at least 3 days earlier or later than expected, women were asked to repeat the AA during the next cycle in order to ensure a late luteal-phase assessment.

Women performed eight assessments per day starting exactly at 9:00 h. The remaining seven assessments took place between 10:00 h and 21:30 h at semi-random time points with a completion time of 3–4 min per assessment. Ignored or rejected alarms were coded as missing (for detailed information see Beddig et al., 2019).

### Ambulatory assessment (AA) variables

Momentary NA and PA were assessed using 12 items from previous AA studies by our group (e.g. Kuehner, Welz, Reinhard, & Alpers, 2017; Timm et al., 2018). At each assessment, women reported the extent to which they felt several negative (upset, irritated, nervous, listless, down, and bored, *α* = 0.832^†^^1^) and positive (cheerful, energetic, enthusiastic, satisfied, relaxed, and calm, *α* = 0.708) emotions on a seven-point Likert scale ranging from 1 (not at all) to 7 (very much).

At the first assessment (09:00 h) of each AA day, women further reported time of awakening and sleep duration (number of hours) as well as sleep quality (‘How did you sleep last night?’) measured on a seven-point Likert scale ranging from 1 (very bad) to 7 (very good) by single items.

### Salivary measure of cortisol

Twenty minutes after each subjective AA, women collected saliva cortisol samples with standard salivettes (Sarstedt, Germany), resulting in eight saliva samples per day for analysis. Women were instructed not to eat, drink anything other than water, smoke, physically exercise, and brush their teeth the next 20 min until saliva collection, and also to refrain from strenuous exercise during AA days (cf. Schlotz, 2019). Immediately after collection of each cortisol sample, they indicated whether they had eaten, drunk anything other than water, smoked, or brushed their teeth (0 = no, 1 = yes) and the extent of their physical activity (seven-point Likert scale: 1 = not at all to 7 = very much) during the last 20 min. The smartphone further provided a random three-digit code, which had to be recorded on the respective label of the salivette tube used during each saliva collection (Schlotz, 2019). Until being returned, all samples were stored in the participants' home freezer and were frozen at −20 °C at the laboratory prior to biochemical analysis. At the laboratory of Professor Kirschbaum (Dresden, Germany), salivettes were centrifuged at 3000 rpm for 5 min, resulting in a clear supernatant of low viscosity. Salivary concentrations were measured using commercially available chemiluminescence-immunoassay with high sensitivity (IBL International, Hamburg, Germany). The intra- and interassay coefficients for cortisol were below 8%.

### Trait-level measures

#### Mindfulness

The German version of the 15-item Mindfulness Attention Awareness Scale (MAAS; Brown & Ryan, 2003) was administered to measure participants' habitual tendency to be attentive and aware of present-moment experiences. The items were rated on a six-point Likert scale, with higher scores indicating greater mindfulness (*α* = 0.891).

#### Reappraisal

The six-item subscale of the Emotion Regulation Questionnaire (ERQ; Abler & Kessler, 2009) was used to assess habitual usage of reappraisal. All items were rated on a seven-point Likert scale with higher scores indicating higher usage of reappraisal (*α* = 0.842).

#### Repetitive negative thinking (RNT)

Participants completed the German version of the Perseverative Thinking Questionnaire (PTQ; Ehring et al., 2011), a well-validated 15-item scale assessing the habitual tendency to engage in RNT. All items were answered on a five-point Likert scale with higher scores signifying higher levels of RNT (*α* = 0.957).

#### Neuroticism

Neuroticism was measured with the 12-item Neuroticism Subscale derived from the NEO Five Factor Inventory (NEO-FFI; Costa & McCrae, 1992; *α* = 0.879).

### Depressive symptoms

The severity of self-rated depressive symptoms during the last 2 weeks was measured with the 21-item Beck Depression Inventory-II (BDI-II; Beck, Steer, & Brown, 1996; *α* = 0.915).

### Statistical analyses

#### Group differences in ER-strategies

To test group differences in habitual ER-strategies, a one-way multivariate analysis of variance (MANOVA) was conducted with group (PMDD *v.* controls) as the independent variable and cognitive ER-strategies (mindfulness, reappraisal, and RNT) as dependent variables.

#### Associations of ER-strategies with daily affect and cortisol in women with PMDD

To examine the predictive value of ER-strategies on the course of momentary affect and cortisol across the menstrual cycle in women with PMDD (*N* = 61), multilevel models (MLM) were estimated to take into account that assessments (level 1) were nested within participants (level 2) (Nezlek, Schroeder-Abé, & Schuetz, 2006). Prior to the main analyses, random intercept models were fitted for each outcome to calculate intraclass correlation coefficients (ICC). Furthermore, possible confounders of daily affect (time since awakening, time since first assessment, assessment day) were analyzed in separate random intercept models and were retained in the models if significant (*p* < 0.05). This applied to assessment day. All level 2 predictors were grand-mean-centered within the PMDD-group to improve the interpretability of the resulting MLM parameters.

Cortisol data were log-transformed to adjust for skewness. Then, outliers more than three standard deviations from the group mean were winsorized to three standard deviations (Stalder et al., 2016). Time was centered at waking time. Possible confounders of basal cortisol secretion (age, current medication use, habitual smoking, time, time^2^, time of awakening, sleep quality, sleep duration, weekday *v.* weekend, and drinking anything other than water, smoking cigarettes, eating, brushing the teeth, and the level of physical activity during the last 20 min) were analyzed in separate random intercept models, and were retained in the models if significant (*p* < 0.05). This applied to time, time^2^, time of awakening, sleep duration as well as recent physical activity and drinking.

Random intercept models, in which the intercept was allowed to vary between individuals, were estimated using restricted maximum likelihood estimation (REML). MLMs were carried out in two steps. First, we estimated the main effects of each habitual ER-strategy (mindfulness, reappraisal, and RNT) on each momentary outcome (NA, PA, cortisol) in separate MLMs and controlled for cycle phase as a categorical level 2 variable. In a second step, these models were expanded by entering the interaction effects of cycle phase with ER-strategies (cycle phase×ER-strategy) on each momentary outcome. In case of a significant interaction effect, we subsequently estimated simple slopes for each ER-strategy on mood and cortisol per cycle phase in post-hoc analyses. Finally, all analyses were repeated by controlling for possible main effects of neuroticism and depressive symptoms.

Statistical analyses were performed using IBM SPSS version 25 with the significance level set at *α* = 0.05. This value was not adjusted for multiple testing as the tests were based on preplanned hypotheses (Armstrong, 2014).

## Results

### Sample description

The descriptives on demographics and questionnaire measures are listed in Table 1. Groups did not significantly differ with respect to age, education level, work situation, partner status, and percentage of having children. However, as expected, women with PMDD displayed higher levels of depressive symptoms and neuroticism scores.

**Table 1.** Demographic and clinical characteristics of women with PMDD and controls

	PMDD (*n* = 61) %/*M* (s.d.)	Controls (*n* = 61) %/*M* (s.d.)	Test statistic	*p*
Demographic variables				
Age	29.4 (5.8)	29.5 (5.1)	*t*(120) = −0.03	0.977
Education (% with high school degree)	72.1%	75.4%	χ^2^(1) = 0.17	0.681
Work situation (% in regular job or education)	80.3%	90.2%	χ^2^(1) = 2.35	0.126
Partner status (% married or living together)	60.7%	59.0%	χ^2^(1) = 0.03	0.853
Children (%)	24.6%	26.2%	χ^2^(1) = 0.04	0.835
BMI	23.6 (4.1)	23.5 (4.3)	*t*(120) = 0.12	0.903
Clinical variables				
SCID-I lifetime diagnosis of depression	54.1%	21.3%	χ^2^(1) = 13.96	<0.001
BDI-II depression score	10.9 (8.9)	4.8 (5.6)	*t*(119) = 4.51	<0.001
NEO-FFI neuroticism	2.11 (0.7)	1.5 (0.7)	*t*(118) = 4.46	<0.001

BMI, body mass index; SCID-I, Structured Clinical Interview for DSM-IV Axis I; BDI-II, Beck Depression Inventory-II; NEO-FFI, NEO Five Factor Inventory.

### Group differences in ER-strategies

The MANOVA yielded a significant effect of group (PMDD *v.* controls) on ER-strategies [Wilks' *λ* = 0.852; *F*_(3,116)_ = 6.71, *p* < 0.001]. Univariate ANOVAs revealed significant group differences in habitual mindfulness [*F*_(1,3)_ = 4.55, *p* = 0.035], reappraisal [*F*_(1,7)_ = 5.31, *p* = 0.023], and RNT [*F*_(1,12)_ = 18.19, *p* < 0.001]. In particular, women with PMDD reported lower levels of mindfulness (*M*_PMDD_ = 4.13, s.d._PMDD_ = 0.96; *M*_controls_ = 4.45, s.d._controls_ = 0.69), less use of reappraisal strategies (*M*_PMDD_ = 4.19, s.d._PMDD_ = 1.34; *M*_controls_ = 4.67, s.d._controls_ = 0.88), and higher levels of RNT (*M*_PMDD_ = 1.92, s.d._PMDD_ = 0.91; *M*_controls_ = 1.29, s.d._controls_ = 0.70).

### Multilevel analyses (MLM)

#### Compliance

MLMs were based on 61 women with PMDD. Altogether, 3381 of 3904 possible subjective assessments (four menstrual cycle phases × 16 assessments per phase × 61 participants) were recorded, which corresponds to a response rate of 86.6%. This reflects a high level of compliance (cf. Courvoisier, Eid, & Lischetzke, 2012). The compliance rate for cortisol assessments in the PMDD group amounted to 84.5%.

#### Intra-class correlation (ICC)

ICCs indicated that 22% of variability in NA and 26% of variability in PA of the PMDD sample were attributable to between-person differences. For cortisol assessments, the ICC indicated that 16% of variability in cortisol levels were due to between-person differences.

#### Associations of ER-strategies with momentary affect across the cycle

The main effect analyses revealed that lower mindfulness [*B* = −0.15, s.e. = 0.07, *t*(59) = −2.30, *p* = 0.025] and reappraisal [*B* = −0.12, s.e. = 0.05, *t*(59) = −2.68, *p* = 0.010], and higher RNT levels [*B* = 0.16, s.e. = 0.07, *t*(59) = 2.27, *p* = 0.027] predicted increased NA across the menstrual cycle (Table 2). In turn, higher mindfulness [*B* = 0.22, s.e. = 0.08, *t*(59) = 2.93, *p* = 0.005] and reappraisal [*B* = 0.15, s.e. = 0.06, *t*(59) = 2.81, *p* = 0.007] and lower RNT levels [*B* = −0.23, s.e. = 0.08, *t*(59) = −2.80, *p* = 0.007] predicted increased PA across the menstrual cycle (Table 2).

**Table 2.** Main and interaction effects of ER-strategies with cycle phase on momentary affect and cortisol activity

Predictor	Negative affect (NA)^a^			Positive affect (PA)^a^			Cortisol^b^		
	df	*F*	p	df	*F*	*p*	df	*F*	*p*
Mindfulness									
Step 1									
Cycle phase	3, 3320	105.30	<0.001	3, 3320	91.19	<0.001	3, 3011	0.87	0.458
Mindfulness	1, 59	5.30	0.025	1, 59	8.60	0.005	1, 59	3.18	0.080
Step 2^c^									
Cycle phase × mindfulness	3, 3317	6.76	<0.001	3, 3317	4.03	0.007	3, 3007	3.09	0.026
Reappraisal									
Step 1									
Cycle phase	3, 3320	105.37	<0.001	3, 3320	91.27	<0.001	3, 3011	0.86	0.462
Reappraisal	1, 58	7.17	0.010	1, 59	7.90	0.007	1, 59	0.51	0.478
Step 2^c^									
Cycle phase × reappraisal	3, 3317	5.37	<0.001	3, 3317	3.21	0.022	3, 3007	2.58	0.052
Repetitive negative thinking (RNT)									
Step 1									
Cycle phase	3, 3320	105.37	<0.001	3, 3320	91.27	<0.001	3, 3010	0.86	0.461
RNT	1, 59	5.16	0.027	1, 59	7.81	0.007	1, 59	0.14	0.712
Step 2^c^									
Cycle phase × RNT	3, 3318	6.97	<0.001	3, 3317	6.17	<0.001	3, 3008	2.06	0.103

ER-strategies, emotion regulation strategies; RNT, repetitive negative thinking.

All models include random intercepts at level 2.

a Models include fixed effects of assessment day.

b Models include fixed effects of time, time^2^, time of awakening, sleep duration as well as physical activity and drinking during the past 20 min.

c Step 2 models additionally include main effects of cycle phase and ER-strategy.

**Fig. 1.**
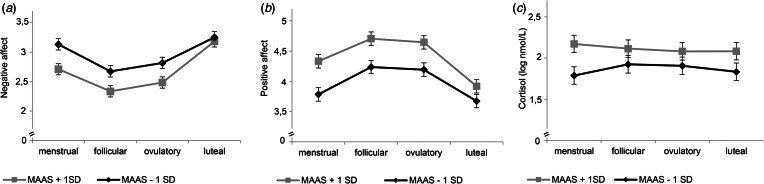
Interaction effects of mindfulness and cycle phase on momentary affect and cortisol activity. MAAS, Mindfulness Attention Awareness Scale. Estimated mean values of momentary negative affect (*a*), positive affect (*b*), and log-transformed basal cortisol activity (*c*) per menstrual cycle phase for low and high scores on MAAS (*M* ± 1s.d.) from multilevel models for illustration purposes. Error bars represent standard error of the estimated mean. All models include random intercepts at level 2. Models in (*a*) and (*b*) include fixed effects of assessment day. The model in (*c*) includes fixed effects of time, time^2^, time of awakening, sleep duration as well as physical activity and drinking during the past 20 min.

**Fig. 2.**
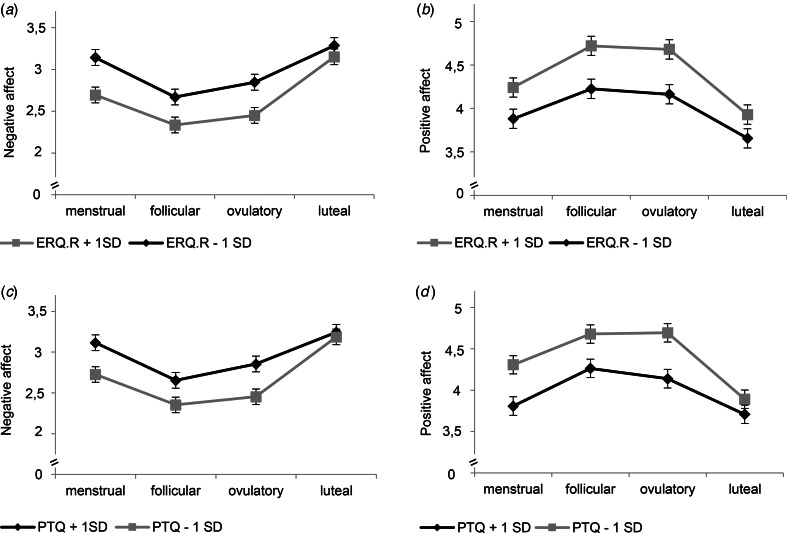
Interaction effects of reappraisal and repetitive negative thinking with cycle phase on momentary affect. ERQ.R, Emotion Regulation Questionnaire_reappraisal subscale; PTQ, Perseverative Thinking Questionnaire. Estimated mean values of momentary negative affect and positive affect per menstrual cycle phase for low and high scores (*M* ± 1s.d.) on ERQ.R (*a*, *b*) and PTQ (*c*, *d*) for illustration purposes. Error bars represent standard error of the estimated mean. All models include random intercepts at level 2 and fixed effects of assessment day.

Our second-step analyses identified significant interaction effects of cycle phase by mindfulness [*F*_NA(3,3317)_ = 6.76, *p* < 0.001; *F*_PA(3,3317)_ = 4.03, *p* = 0.007], reappraisal [*F*_NA(3,3317)_ = 5.37, *p* < 0.001; *F*_PA(3,3317)_ = 3.21, *p* = 0.022], and RNT [*F*_NA(3,3318)_ = 6.97, *p* < 0.001; *F*_PA(3,3317)_ = 6.17, *p* < 0.001] in predicting NA and PA (Table 2). As depicted in Figs. 1 and 2 for illustration purposes, post-hoc tests revealed that favorable ER-strategies, i.e. higher mindfulness and reappraisal and lower RNT levels, predicted decreased NA and increased PA only in the menstrual, follicular, and ovulatory phases (all *p*s < 0.029, see Table 3 for post-hoc test results), while these ER-strategies did not show any effect on momentary affect in the late luteal phase (all *p*s > 0.05). Thus, contrary to our hypotheses, women with favorable ER-strategies showed stronger mood deterioration toward the late luteal phase, thereby converging with women with unfavorable ER-strategies (see Figs. 1 and 2).

**Table 3.** Estimated simple slopes of mindfulness, reappraisal, and repetitive negative thinking (RNT) on negative affect, positive affect, and cortisol activity per cycle phase

ER-strategy	Cycle phase	Negative affect		Positive affect		Cortisol^a^	
		*B* (s.e.)	*p*	*B* (s.e.)	*p*	*B* (s.e.)	*p*
Mindfulness	Menstrual phase	−0.22 (0.07)	0.002	0.29 (0.08)	<0.001	0.20 (0.08)	0.012
	Follicular phase	−0.18 (0.07)	0.014	0.25 (0.08)	0.003	0.10 (0.08)	0.201
	Ovulatory phase	−0.17 (0.07)	0.017	0.24 (0.08)	0.005	0.09 (0.08)	0.243
	Luteal phase	−0.04 (0.07)	0.619	0.13 (0.08)	0.120	0.13 (0.08)	0.096
Reappraisal	Menstrual phase	−0.17 (0.05)	0.001	0.14 (0.06)	0.024	–	–
	Follicular phase	−0.13 (0.05)	0.014	0.19 (0.06)	0.002	–	–
	Ovulatory phase	−0.15 (0.05)	0.004	0.19 (0.06)	0.001	–	–
	Luteal phase	−0.05 (0.05)	0.306	0.10 (0.06)	0.085	–	–
Repetitive negative thinking	Menstrual phase	0.22 (0.08)	0.005	−0.28 (0.09)	0.002	–	–
	Follicular phase	0.17 (0.08)	0.029	−0.23 (0.09)	0.010	–	–
	Ovulatory phase	0.22 (0.08)	0.004	−0.31 (0.09)	<0.001	–	–
	Luteal phase	0.03 (0.07)	0.675	−0.10 (0.09)	0.248	–	–

ER-strategy, emotion regulation strategy.

a Log-transformed cortisol values in nmol/l. Simple slope values are presented only for significant interaction terms of cycle phase by ER-strategy.

#### Associations of ER-strategies with momentary cortisol activity across the cycle

The main effects of mindfulness [*B* = 0.13, s.e. = 0.07; *t*(59) = 1.78, *p* = 0.080], reappraisal [*B* = 0.04, s.e. = 0.05; *t*(59) = 0.72, *p* = 0.478], and RNT [*B* = −0.03, s.e. = 0.08; *t*(59) = −0.37, *p* = 0.712] on momentary cortisol activity were not significant (Table 2). However, there was a significant interaction effect of cycle phase and mindfulness on momentary cortisol activity [*F*_(3,3007)_ = 3.09; *p* = 0.026]. As shown in Fig. 1*c*, again for illustration purposes, lower mindfulness was associated with decreased basal cortisol activity only in the menstrual phase, with no associations in the follicular, ovulatory, and luteal phase (see Table 3).

#### Confounder analysis

Neuroticism and severity of depressive symptoms (BDI-II) as covariates did not change the size of any reported ER-strategy × cycle phase interaction effects.

## Discussion

ER deficits are transdiagnostic risk factors and important treatment targets for a variety of psychological disorders (Aldao, Nolen-Hoeksema, & Schweizer, 2010). Unfavorable ER-strategies may interact with the menstrual cycle to contribute to cycle-related changes in mood and cortisol activity in women with PMDD. The present study aimed to compare cognitive ER-strategies in women with and without PMDD, and to explore the predictive value of these strategies for momentary affect and basal cortisol activity across the menstrual cycle in women with PMDD. As expected, women with PMDD reported more maladaptive and less adaptive strategies than healthy controls. Favorable strategies were generally linked to better mood in women with PMDD, whereas they did not appear to protect affected women from cycle-dependent mood worsening during the late luteal phase. Habitual mindfulness was associated with higher cortisol levels during the menstrual phase.

### Differences in the use of ER-strategies between women with and without PMDD

In our study, women with PMDD showed lower mindfulness and reappraisal and higher RNT levels than controls. This is consistent with previous research (Craner et al., 2014; Dawson et al., 2018; Lustyk et al., 2011; Wu et al., 2016), indicating heightened vulnerability toward unfavorable ER-strategies in affected women.

### ER-strategies × cycle phase on momentary affect in women with PMDD

In women with PMDD, higher habitual mindfulness and reappraisal and lower RNT predicted lower NA and higher PA during the menstrual, follicular, and ovulatory phases, but not during the luteal phase. Women reporting more favorable ER-strategies displayed larger mood cyclicity with stronger mood worsening toward the luteal phase, thereby converging with those using more unfavorable strategies. Thus, contrary to our expectations, favorable habitual cognitive ER-strategies seem not to protect affected women from cycle-related mood worsening.

These results may provide first indications of possible limitations of cognitive behavioral interventions (CBI) addressing cognitive ER-strategies in the treatment of PMDD and underscore the need for more nuanced research on possible differential effects of specific interventions. Previous reviews found mixed evidence for CBIs in the treatment of premenstrual dysphoric symptoms (Kleinstäuber, Witthöft, & Hiller, 2012; Lustyk, Gerrish, Shaver, & Keys, 2009). A recent review (Han, Cha, & Kim, 2019) showed that, in particular, the acquisition of active behavioral coping strategies were helpful in premenstrual symptom relief. However, the trials included in these reviews suffer from inadequate study designs and inclusion of women with PMS. A first trial specifically on women with PMDD (Hunter et al., 2002) again showed that the use of active behavioral strategies at the end of a CBI, but not changes in causal attributions, predicted a good clinical outcome after 1 year. A recent 8-week internet-based CBI trial on women with PMDD, which consisted of psychoeducation and cognitive and behavioral strategies, revealed high effect sizes for symptom reduction and psychosocial functioning (Weise et al., 2019). While the effects of specific strategies cannot be extracted from this multimodal intervention, the authors showed that habitual active coping with premenstrual symptoms predicted improved treatment outcomes. Altogether, these results point to potential incremental effects of behavioral over cognitive strategies in PMDD treatment, and may also explain the lack of luteal-phase effects of cognitive ER-strategies on mood in the present study. Evidence-based therapies targeting behavioral skills such as Dialectical-Behavioral-Therapy have already been suggested, but not yet evaluated for PMDD treatment (Eisenlohr-Moul, 2019). However, since we investigated trait characteristics of ER-strategies and not respective interventions, we are aware that these conclusions are highly speculative, but can be tested, for example, in studies with dismantling designs (Papa & Follette, 2015). On the other hand, it is quite conceivable that higher levels of favorable ER-strategies do not necessarily imply that women are able to use these strategies effectively in the premenstrual phase in which mainly biologically determined mood changes occur. The lacking predictive value of habitual ER-strategies for mood-related changes during the luteal phase may also indicate that the unilateral classification of ER in adaptive *v.* maladaptive may be reductive and may ignore the dynamic nature of ER (Aldao, 2013). Instead, the affective impact of ER-strategies may depend on the flexibility to apply them in accordance with contextual demands and individuals' regulatory goals (Aldao, Sheppes, & Gross, 2015; Mikkelsen, Tramm, Zachariae, Gravholt, & O'Toole, 2021; Wenzel, Rowland, Weber, & Kubiak, 2020). In PMDD, contextual, especially cycle-phase-specific, characteristics may predict the choice and the efficacy of ER-strategies (cf. Aldao, 2013; Sheppes, Scheibe, Suri, & Gross, 2011; Wenzel, Rowland, & Kubiak, 2021). In this context, AA is particularly suited to assess the possible cycle-phase-specific use of certain ER-strategies at the state level. Furthermore, it offers the opportunity to directly induce specific ER-states and to assess their cycle-specific effects in an experimental field design (cf. Huffziger et al., 2013 for a similar approach; Huffziger, Ebner-Priemer, Koudela, Reinhard, & Kuehner, 2012). In this way, future research can gain a deeper understanding of ER-processing and its impact on affect across the menstrual cycle in women with PMDD.

### ER-strategies × cycle phase on momentary cortisol activity in women with PMDD

To our knowledge, our study is the first to investigate the impact of ER-strategies on cycle-related variations of basal cortisol activity in women with PMDD. Mindfulness, reappraisal and RNT did not predict overall or luteal-phase-specific cortisol output. In contrast, higher trait mindfulness was linked to higher cortisol levels in the menstrual phase.

The high intra-individual variability of cortisol release in the current study (ICC = 16%) matches with the observation that substantial variance in diurnal cortisol is due to moment-to-moment and day-to-day fluctuations rather than to stable between-person differences (Doane, Chen, Sladek, Van Lenten, & Granger, 2015). In experimental studies inducing mental stress, women with affective disorders and women with PMDD show blunted cortisol activity (Hantsoo & Epperson, 2015; Zorn et al., 2017). In contrast, no clear associations have been identified between induced ER-strategies and cortisol responses in general (Mikkelsen et al., 2021). The effects of trait ER-strategies on diurnal cortisol levels outside the lab have also only been rarely examined (Otto, Sin, Almeida, & Sloan, 2018).

The lacking effect of trait reappraisal on daily life cortisol is in line with results from nonclinical samples (Otto et al., 2018; Rnic, Jopling, Tracy, & LeMoult, 2022) and has been attributed to the low physiological effort required for reappraisal as a more automatic process in the long term (Otto et al., 2018). There is also some indication that reappraisal is more effective in the context of controllable stressors (cf. Mikkelsen et al., 2021). Given that women with PMDD often describe themselves as feeling out of control during the late luteal phase (cf. APA, 2013), this may explain why this ER-strategy may not be able to affect cycle-related cortisol activity. Similarly, the lacking effect of trait RNT on cortisol is in accordance with findings, which show that state measures of RNT are more closely related to cortisol activity than trait measures, particularly if the latter do not specifically measure stress-related RNT (Zoccola & Dickerson, 2012).

High habitual mindfulness appeared to counteract low cortisol secretion particularly in the menstrual phase, but not in the luteal phase (as expected). Blunted presentation of cortisol secretion seems to be associated with a triad of increased pain, stress sensitivity, and fatigue (Fries, Hesse, Hellhammer, & Hellhammer, 2005), and higher cortisol concentrations are commonly related to lower pain intensity (cf. Ubeda-D'Ocasar et al., 2020). In turn, studies have shown beneficial effects of mindfulness-based interventions on menstrual pain and pain perception (Payne, Seidman, Romero, & Sim, 2020; Purnamasari, Rohita, Zen, & Ningrum, 2020), and the activation of cortisol release may constitute one possible mechanism through which habitual mindfulness can dampen respective symptoms. However, these considerations are again speculative because we did not measure cycle-related physical symptoms in our study, which is clearly warranted in future AA studies.

### Limitations and future directions

Our study has some limitations. First, the sample size was modest although exceeding the recommended minimum size for estimating cross-level interactions (Hox, Moerbeek, & Van de Schoot, 2017). Second, in order to keep participants' burden low and compliance rates high within this intensive AA-design, the PMDD diagnosis was assessed with a retrospective, although well-validated, structured interview (SCID-PMDD; Accortt et al., 2011). This in turn can bear the risk of recall-bias toward false-positive symptom reports (Schmalenberger et al., 2021). DSM-5 requires prospective daily symptom ratings for at least two symptomatic cycles for a definite diagnosis. Thus, the present PMDD-diagnoses must be considered provisional (APA, 2013).

Furthermore, we only examined cognitive ER-strategies. The further inclusion of behavioral ER-strategies (e.g. stress-reduction-skills) would allow to assess possible effects of specific classes of strategies. We also focused on measures of self-assessed habitual ER-strategies, which are characterized by possible recall-bias, restriction to conscious ER, and by unclear predictive validity regarding their use in daily life (Naragon-Gainey et al., 2017). In this context, implicit assessments of ER (e.g. Eggert et al., 2017) and AA-designs can help to uncover automatic aspects of ER-processes and possible cycle-specific ER-deficits and, consequently, possible treatment targets.

## Conclusions

The present findings suggest that protective habitual cognitive ER-strategies are generally linked to improved momentary mood in women with PMDD but do not appear to protect affected women from cycle-dependent mood deteriorations. Conversely, habitual mindfulness seems to exert a beneficial effect on basal cortisol activity only in the menstrual phase. These findings emphasize the importance of a refined research on ER and its interaction with cycle phases to predict psychological and endocrinological changes in PMDD across the menstrual cycle. This will also help the progression towards an individualization of PMDD treatment targets.

## Footnotes

† The notes appear after the main text.

## Note

1 All reported Cronbach's alpha values (α) in the paper refer to the present sample.
